# Reduced Recurrence Rates Are Associated with Photodynamic Diagnostics Compared to White Light after Extended Transurethral Resection of Bladder Tumors

**DOI:** 10.3390/life12050641

**Published:** 2022-04-26

**Authors:** Alexander Marquardt, Mario Richterstetter, Helge Taubert, Arndt Hartmann, Bernd Wullich, Verena Lieb, Laura Bellut, Sven Wach, Hendrik Apel

**Affiliations:** 1 Department of Urology and Pediatric Urology, University Hospital Erlangen, Friedrich-Alexander Universität Erlangen, Krankenhausstrasse 12, 91054 Erlangen, Germany; alexander.marquardt@hotmail.de (A.M.); mario.richterstetter@gmail.com (M.R.); helge.taubert@uk-erlangen.de (H.T.); bernd.wullich@uk-erlangen.de (B.W.); verena.lieb@uk-erlangen.de (V.L.); laura.bellut@uk-erlangen.de (L.B.); hendrik.apel@uk-erlangen.de (H.A.); 2 Comprehensive Cancer Center Erlangen-EMN (CCC ER-EMN), 91054 Erlangen, Germany; arndt.hartmann@uk-erlangen.de; 3 Institute of Pathology, University Hospital Erlangen, Friedrich-Alexander Universität Erlangen, Krankenhausstrasse 8-10, 91054 Erlangen, Germany

**Keywords:** transurethral resection, photodynamic diagnostics, recurrence-free survival

## Abstract

One pillar in treating non-muscle-invasive bladder cancer (NMIBC) is the complete and high-quality transurethral resection of the primary tumor (TURBT). However, even after a high-quality primary resection, the residual tumor risk is considerable, thus requiring a re-TURBT. Resections performed with the aid of a photodynamic diagnostics report improved recurrence-free survival rates and increased detection rates of carcinoma in situ (CIS). This monocentric retrospective study reports on patients treated with an extended TURBT procedure using conventional white-light cystoscopy or photodynamic diagnostics (PDD). Only patients undergoing a TURBT resection for their primary tumor were included in the statistical analysis. Recurrence-free survival and overall survival were the clinical endpoints. Mann–Whitney U tests and chi-squared tests were used for descriptive intergroup comparisons. The associations with overall survival and recurrence-free survival were determined by univariate and multivariate analyses. The test results were considered significant when *p* was < 0.05. In comparison to conventional white-light cystoscopy, PDD increased the detection rates of CIS (*p* = 0.004) and tumor multifocality (*p* = 0.005) and led to reduced residual tumor incidence at the primary resection site (*p* < 0.001). Likewise, tumor recurrence rates were reduced in the PDD cohort (*p* < 0.001). Patient age and the presence of residual tumor at the primary resection site were identified as independent predictors of overall survival. For recurrence-free survival, only the PDD resection method was an independent predictor (HR = 0.43; *p* < 0.001). In summary, we demonstrated that the utilization of PDD techniques was associated with improved detection rates of CIS and multifocal tumors and with reduced recurrence rates. The extended resection protocol allowed us to determine that PDD resections lead to a reduced residual tumor rate at the initial resection site. This residual tumor state at the resection site, determined by extended TURBT, became an independent predictor of long-term survival. On the other hand, the PDD technique was confirmed as the only independent predictor of recurrence-free survival.

## 1. Introduction

Urothelial cancer of the bladder (BCA) represents a major source of cancer-related morbidity and mortality. More than 570,000 new cases are diagnosed worldwide per year. BCA’s age-standardized incidence rates (per 100,000 individuals) account for 9.5 in males and 2.4 in females, making it the 12th most common cancer [1]. At initial diagnosis, approximately 75% of patients present with non-muscle-invasive bladder cancer (NMIBC) [2], which allows for organ-sparing therapy approaches. Both the treatment guidelines of the European Association of Urology (EAU) [3] and the guidelines of the American Urological Association (AUA) [4] recommend transurethral resection of the bladder tumor (TURBT) as the standard treatment. Fractionated or en bloc resection methods are both valid and approved [5].

There is a broad consensus that a complete resection is essential for a good prognosis. Thus, the surgeons’ professional experience in performing the resection has been described as an independent prognostic factor for a longer recurrence-free survival [6]. Moreover, histopathological characteristics, such as the presence of detrusor muscle in the resection specimen, may serve as a proxy marker for complete resection and are associated with a reduced risk of recurrence [7,8]. Other risk factors for recurrence and progression include tumor multifocality, tumor size, tumor stage, concomitant carcinoma in situ (CIS), and tumor grading [9,10].

Nevertheless, even after a well-performed TURBT, there is still a considerable risk of residual tumor [11]. Therefore, a second resection (re-TURBT) is recommended within six to eight weeks after the primary resection [12] to further increase the recurrence-free survival of patients [13]. The rates of residual tumor in re-TURBT resections can reach up to 55–60% [14].

However, even after an experienced provider TURBT procedure and intravesical BCG instillation, approximately 35% of patients experience a local disease recurrence within 3 years [15].

In 2008, the method of photodynamic diagnostics (PDD) was approved by the EAU, and a systematic review of prospective studies described better diagnostic accuracy with fluorescent methods (additional tumor lesions detected and higher CIS detection rates) as well as improved recurrence-free survival [16]. The approved fluorescent agent, hexaminolaevulinate (HAL), exhibits the same diagnostic capabilities as 5-aminolaevulinic acid (ALA) [17]. Although the diagnostic benefit of PDD has been confirmed multiple times, it has recently been demonstrated that, in German-speaking countries, the majority (60%) of urologists in the outpatient setting perform only white-light cystoscopies [18].

In this monocentric series, we analyzed the clinical and demographical parameters of patients with NMIBC treated with TURBT for the resection of their primary tumor, performed with or without the help of PDD. The influence of the PDD procedure on the detection rates of CIS and tumor multifocality was analyzed. Additionally, the impact of PDD on patient overall survival and recurrence-free survival was analyzed in comparison to our historical cohort, where only conventional white-light resection was applied. We have previously reported establishing an extended TURBT technique that incorporates a series of additional ground and margin specimens to better evaluate the quality of the initial resection [19]. This extended TURBT technique allowed us to additionally assess the impact of the PDD procedure upon the presence of residual tumor in extended deep and margin specimens.

## 2. Materials and Methods

We retrospectively reviewed a total of 881 TURBT resections that were performed in our institution between 1986 and 2014, following an extended TURBT protocol that was thoroughly described in an earlier publication [19]. Extended TURBT under conventional white-light conditions was performed between 1986 and 2013 using monopolar instrumentation, and extended TURBT under PDD conditions was performed between 2003 and 2014 using bipolar instrumentation. For PDD, the patients received an intravesical instillation with 8 mM hexaminolaevulinate (HAL; HEXVIX) for one hour before planned surgery. PDD was performed intra-surgical and as a final control using a TriCam SL system (Storz medical, Tägerwilen, Switzerland) and a D-LightC illumination source (Storz). After completing the TUR resection of the primary tumor, all patients underwent the extended TURBT protocol. For this, additional specimens were taken from the center of the resection area (1 to 4 samples, depending on the size of the primary tumor) and from the normal-appearing urothelium at the margin of the resection area (3 to 4 samples). All specimens were reviewed by an experienced uro-pathologist. The pathological results of the additional specimens were used to determine the resection margin status (sR status).

All patients, irrespective of the primary resection method, underwent a scheduled re-TURBT within 6–8 weeks of the primary resection using conventional white-light cystoscopy and were further treated according to current clinical guidelines and best clinical practice protocols. This also included supportive instillation therapies with mitomycin C or Bacillus Calmette-Guérin (BCG) at the surgeon’s discretion. Patient follow-up was conducted by regular cystoscopic examination using conventional white-light technology.

All patients, starting in 2008, provided written informed consent. For samples collected before 2008, the Ethics Committee in Erlangen waived the need for informed individual consent. The study was approved by the Ethics Committee of the University Hospital Erlangen (No. 3755). The patients’ clinical, pathological, and long-term follow-up information was retrieved from the tumor registry of the Comprehensive Cancer Center Erlangen-EMN (CCC ER-EMN).

Differences in the distribution of parameters between the TURBT with and without PDD were analyzed using chi-square (factor variables) or nonparametric Mann–Whitney (continuous variables) statistical tests. The associations of the clinical parameters and TURBT with overall survival (OS) and recurrence-free survival (RFS) were determined by univariate (Kaplan–Meier analysis and Cox’s regression hazard models) and multivariate analyses (Cox’s regression hazard models). Follow-up intervals were defined as the time from the initial tumor diagnosis to an event (death or recurrence) or the last available patient information. The follow-up intervals for both overall survival and recurrence-free survival were restricted to 120 months, and individuals without events were censored at this time point. A *p* value < 0.05 was considered statistically significant. All calculations were performed with the R statistical framework Ver. 3.2.1 (R Foundation for Statistical Computing, Vienna, Austria. http://www.R-project.org/).

## 3. Results

The clinical records of 881 patients with TURBTs were retrospectively reviewed. The demographical and clinicopathological parameters are presented in Table 1. We excluded 49 re-TURBT specimens and 250 resections of local recurrences from further analyses. Of the remaining 582 primary resections, 116 were classified as muscle-invasive bladder cancer (pT2-pT4) after pathological review or were without documented tumor stage, resulting in a final analysis cohort of 466 cases with a pathological diagnosis of non-muscle-invasive bladder cancer (pTa, pT1, carcinoma in situ). In this final analysis cohort, 219 patients underwent a TURBT under conventional white-light conditions and 247 under PDD conditions.

All patients underwent an extended TURBT that involved taking additional samples from the center and margins of the resection area. In a total of 219 patients, a conventional, white-light cystoscopy approach was used, while in 247 patients, the PDD approach was utilized. Patients in the PDD subgroup were older, and a higher proportion of women were treated using this technique (Table 2).

Regarding tumor-specific characteristics, it was evident that extended TURBT with PDD was significantly associated with a higher detection rate of high-grade tumors, particularly G3, concomitant CIS, and tumor multifocality (Table 2). As determined by the pathological review of the additional specimens (sR status), the resection margin status demonstrated significantly fewer sR1 conditions. Consequently, as the PDD approach was associated with an increased diagnostic detection rate of high-risk tumors (concomitant CIS, high-grade G3 tumors, and multifocality), we also observed a significantly increased rate of supportive instillation therapy (Table 2). Regarding the recurrence and survival status of the patients, the PDD technique was associated with a lower rate of tumor recurrence, while no impact on overall survival was observed.

We then tested for an association of the parameters with patients’ overall survival (Table 3). In univariate analyses, the parameters of patient age (HR = 1.08), positive sR1 resection (HR = 2.03), tumor multifocality (HR = 1.48), and BCG instillation (HR = 0.53) were associated with overall survival (all *p* < 0.05). In a multivariate model incorporating all parameters, the patient’s age (HR = 1.07; *p* < 0.001) and positive sR1 resection margins (HR = 2.54; *p* = 0.001) were identified as independent predictors of patient survival (Table 3).

Finally, we tested for the association of the parameters with patients’ recurrence-free survival (Table 4). In univariate analyses, only the parameters of patient age (HR = 0.99) and the resection procedure were associated with recurrence-free survival, with the PDD patient group exhibiting a 0.42-fold risk of tumor recurrence.

In a multivariate model incorporating all parameters, extended TURBT with PDD (HR = 0.43; *p* < 0.001; Table 4) was confirmed as the only independent predictor of recurrence-free survival. In contrast to overall survival, resection status was not a predictor of recurrence-free survival. To test whether our comparison of a historic non-PDD cohort with a recent PDD cohort introduced a relevant selection bias, we followed described methods [20] and performed bootstrap resampling followed by automated variable selection methods. Based on 1,000 independent bootstrap replicates, we found that in 96.7% of the replicates, the resection procedure (Conventional white-light or PDD) was retained as an independent predictor of recurrence-free survival, thus arguing against a relevant selection bias.

**Figure 1:**
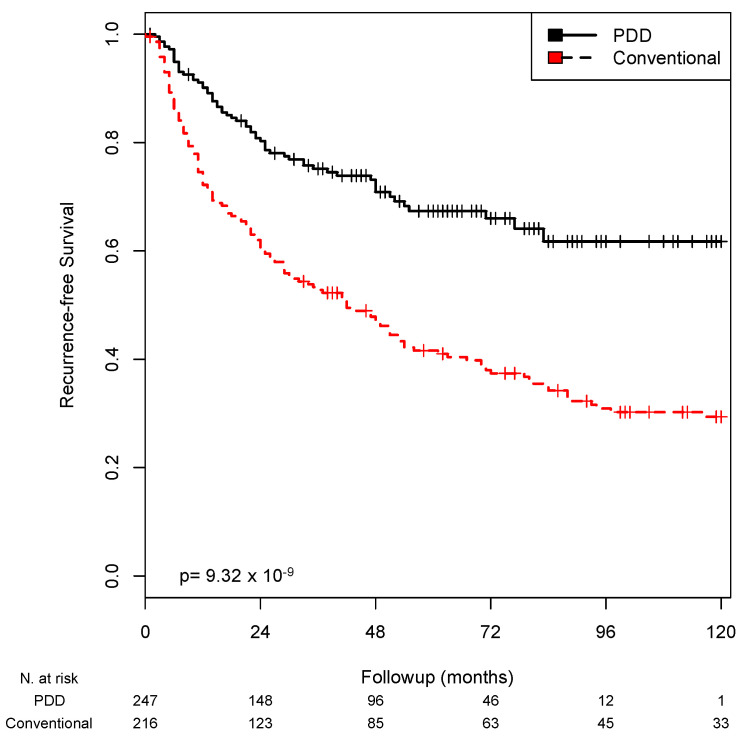
Kaplan–Meier analysis. The patients were stratified according to whether PDD was used for extended TURBT. The recurrence-free survival rates are shown (*p* < 0.001).

A Kaplan–Meier analysis of recurrence-free survival demonstrated that patients undergoing TURBT without PDD had a mean time to local recurrence of 57 months. For patients undergoing TURBT with PDD, this interval was 86 months (*p* < 0.001; Figure 1).

## 4. Discussion

In the clinical management of non-muscle-invasive bladder cancer (NMIBC), one pillar of clinical treatment is the transurethral resection of the tumor. There is broad consensus that a high-quality and complete resection is the best prognostic factor for recurrence-free survival and long-term outcome [6,21]. Several attempts have been conducted to predict patients’ recurrence-free survival and long-term outcome, which resulted in different risk score calculators [9,22,23,24].

Besides the morphological and histological characteristics of the cancer itself or peri-operative treatment protocol, only a few parameters described are associated with recurrence-free survival. These include factors such as the surgeon’s personal experience [6] or the presence of detrusor musculature in the resection specimen [7,8]. Likewise, immediate peri-operative instillation therapies such as mitomycin C can reduce recurrence rates, especially in patients with an EORTC risk score below five [25]. Additionally, a re-TURBT 6-8 weeks after the initial resection is suggested, and it has been estimated that omitting re-TURBT would lead to a 44% higher risk of recurrence [26].

New diagnostic methods, such as photodynamic diagnostics, have improved recurrence-free survival rates along and allowed higher detection rates of multifocality and CIS [16]. The diagnostic advantage of PDD is a well-established fact [27]. It has been reported that approximately 13% more pT1 lesions were detected only because of the increased sensitivity of the PDD procedure [28]. However, the impact of TURBT resections with PDD on patients’ long-term recurrence-free survival remained uncertain until a recent observational study [29] and a meta-analysis [30] confirmed improved long-term recurrence-free survival with PDD resections.

We have previously described the technique of an extended TURBT protocol that showed that residual tumor rate, particularly at the resection margin, could be as high as 30% [19]. In the current study, we analyzed the clinical and demographical parameters of patients with NMIBC treated with the described extended TURBT protocol under conventional white-light or PDD conditions.

The diagnostic characteristics of PDD resections in our cohort are in accordance with previous publications that report higher detection rates of T1 tumors, high-grade tumors, and multifocal tumors [29,30]. Although an increased CIS detection rate has been described [16,30], we discovered a very high rate (26%) of CIS detection under PDD conditions.

Some studies have already described that TURBT resections using PDD are associated with improved recurrence-free survival [29,31,32]. However, differences exist between the various studies that are related to the treatment protocol (no adjuvant treatment: Miyake et al. [32]) or the lack of available information about re-TURBT [31]. This complicates a direct comparison.

Our study represents one of the most extensive monocentric observational studies. In contrast to the patient cohort studied by Gallagher et al. [29], where all patients received early supportive instillation therapy within 24 hours, and only 80% of high-risk patients underwent re-TURBT, all of our patients underwent scheduled re-TURBT, and 56% received supportive instillation therapy according to the current guidelines. However, in our patient cohort, the recurrence rates, especially in resection with PDD (25% overall recurrence), were below those reported in [29].

In multivariate regression analyses, a resection using PDD was the only independent predictor of recurrence-free survival. This again supports the assumption that the detection of adverse histological findings (CIS, high tumor grade, and tumor multifocality) and the subsequent administration of supportive therapy are highly dependent on the diagnostic procedure.

Thus far, it is unclear whether a tumor resection performed with or without PDD impacts overall survival. One randomized study reported no significant difference in overall survival depending on the resection procedure [33]. In addition to patient age, we showed that the sR status, determined by taking additional sample specimens, was the only independent predictor of patient overall survival in our patient set. This again supports the position that complete resection of the primary tumor is the most important predictor of long-term survival.

The major limitation of our study is the comparison of the PDD cohort with a historic non-PDD cohort, which may lead to a particular bias regarding patient selection, change in treatment regimens, or improvement in histopathologic processing. Likewise, the technical equipment changed over time, with the conventional white-light TURBT being performed with monopolar resection equipment and the TURBT under PDD conditions with bipolar equipment. Nevertheless, our results of multivariate outcome regression indicate that in NMIBC, the utilization of PDD in conjunction with extended TURBT is associated with improved detection rates of high-grade tumors, CIS, and multifocality. The reduced recurrence rate demonstrated here may be explained by an improved primary resection leading to a higher number of tumor-free resection margins or by a more accurate grading and staging of the tumors resulting in a higher number of supportive instillation therapies or both.

## Figures and Tables

**Table 1:** Clinicopathological and prognostic data.

Parameter	All Patients	Primary Resection	Recurrence	re-TURBT
Procedure, *n* (%)				
Conventional white light	435 (49.4)	288 (49.5)	118 (47.2)	29 (59.2)
PDD	446 (50.6)	294 (50.5)	132 (52.8)	20 (40.8)
Age, years (IQR)	70 (61–77)	70 (60–77)	69 (61–77)	74 (65–80)
Sex, *n* (%)				
Female	185 (21.0)	134 (23.0)	45 (18.0)	6 (12.2)
Male	696 (79.0)	448 (77.0)	205 (82.0)	43 (87.8)
Tumor stage, *n* (%)				
Ta	457 (51.9)	310 (53.3)	132 (52.8)	15 (30.6)
Tis/T1	224 (25.4)	156 (26.8)	48 (19.2)	20 (40.8)
T2–T4	142 (16.1)	108 (18.5)	22 (8.8)	12 (24.5)
n.a.	58 (6.6)	8 (1.4)	48 (19.2)	2 (4.1)
Tumor grading, *n* (%)				
G1	209 (23.7)	139 (23.9)	62 (24.8)	8 (16.3)
G2	276 (31.3)	194 (33.3)	75 (30.0)	7 (14.3)
G3	340 (38.6)	241 (41.4)	67 (26.8)	32 (65.3)
n.a.	56 (6.4)	8 (1.4)	46 (18.4)	2 (4.1)
Resection margins, *n* (%)				
sR0	530 (60.2)	394 (67.7)	109 (43.6)	27 (55.1)
sR1	181 (20.5)	141 (24.2)	27 (10.8)	13 (26.5)
sRx	32 (3.6)	30 (5.2)	2 (0.8)	0 (0.0)
n.a.	138 (15.7)	17 (2.9)	112 (44.8)	9 (18.4)
CIS, *n* (%)				
Not detected	613 (69.6)	435 (74.7)	155 (62.0)	23 (46.9)
Detected	207 (23.5)	135 (23.2)	46 (18.4)	26 (53.1)
n.a.	61 (6.9)	12 (2.1)	49 (19.6)	0 (0.0)
Tumor focality, *n* (%)				
Unifocal	500 (56.8)	385 (66.2)	98 (39.2)	17 (34.7)
Multifocal	326 (37.0)	184 (31.6)	111 (44.4)	31 (63.3)
n.a.	55 (6.2)	13 (2.2)	41 (16.4)	1 (2.0)
Tumor size, *n* (%)				
≤2 cm	250 (28.4)	151 (26.0)	85 (34.0)	14 (28.6)
>2 cm ≤4 cm	156 (17.7)	123 (21.1)	27 (10.8)	6 (12.3)
>4 cm ≤6 cm	63 (7.1)	56 (9.6)	6 (2.4)	1 (2.0)
>6 cm	13 (1.5)	10 (1.7)	2 (0.8)	1 (2.0)
n.a.	399 (45.3)	242 (41.6)	130 (52.0)	27 (55.1)
Instillation, *n* (%)				
None	257 (29.2)	257 (44.2)	0 (0.0)	0 (0.0)
BCG	141 (16.0)	78 (13.4)	52 (20.8)	11 (22.4)
Mitomycin	314 (35.6)	240 (41.2)	65 (26.0)	9 (18.4)
n.a.	169 (19.2)	7 (1.2)	133 (53.2)	29 (59.2)
Recurrence, *n* (%)				
No recurrence	554 (62.9)	345 (59.3)	169 (67.6)	40 (81.6)
Recurrence	319 (36.2)	233 (40.0)	79 (31.6)	7 (14.3)
n.a.	8 (0.9)	4 (0.7)	2 (0.8)	2 (4.1)
Survival, *n* (%)				
Alive	583 (66.2)	388 (66.7)	173 (69.2)	22 (44.9)
Deceased	291 (33.0)	190 (32.6)	76 (30.4)	25 (51.0)
n.a.	7 (0.8)	4 (0.7)	1 (0.4)	2 (4.1)

**Table 2:** Comparison of clinicopathological and prognostic data between the two groups.

Parameter	Conventional White Light (*n* = 219)	PDD (*n* = 247)	*p* Value
Age years, median (IQR)	67 (58–74)	71 (64–79)	<0.001
Sex, *n* (%)			0.014
Female	40 (18.3)	69 (27.9)	
Male	179 (81.7)	178 (72.1)	
Tumor stage, *n* (%)			0.004
Ta	131 (59.8)	179 (72.5)	
Tis/T1	88 (40.2)	68 (27.5)	
Tumor grading, *n* (%)			0.021
G1	76 (35.5)	63 (25.5)	
G2	86 (40.2)	99 (40.1)	
G3	52 (24.3)	85 (34.4)	
sR, *n* (%)			<0.001
sR0	167 (78.0)	181 (74.8)	
sR1	46 (21.5)	34 (14.0)	
sRX	1 (0.5)	27 (11.2)	
CIS, *n* (%)			0.004
Not detected	182 (84.7)	182 (73.7)	
Detected	33 (15.3)	65 (26.3)	
Tumor focality, *n* (%)			0.005
Unifocal	159 (74.3)	153 (61.9)	
Multifocal	55 (25.7)	94 (38.1)	
Tumor size, *n* (%)			0.093
≤2 cm	80 (60.2)	65 (48.5)	
>2 cm ≤4 cm	36 (27.1)	51 (38.1)	
>4 cm ≤6 cm	15 (11.3)	18 (13.4)	
>6 cm	2 (1.5)	0 (0)	
Instillation therapy, *n* (%)			<0.001
Mitomycin	88 (40.7)	137 (55.5)	
BCG	24 (11.1)	50 (20.2)	
None	104 (48.2)	60 (24.3)	
Recurrence status, *n* (%)			<0.001
No recurrence	79 (36.6)	185 (74.9)	
Recurrence	137 (63.4)	62 (25.1)	
Survival status, *n* (%)			0.107
Alive	150 (69.4)	188 (76.1)	
Deceased	66 (30.6)	59 (23.9)	

**Table 3:** Univariate and multivariate models for overall survival.

Univariate Models for Overall Survival		
Parameter	HR (95% CI)	*p* value
Procedure		
Conventional	1 (Reference)	n.a.
PDD	1.604 (1.109–2.321)	0.012
Age, years	1.080 (1.058–1.104)	<0.001
Sex		
Female	1 (Reference)	n.a.
Male	0.762 (0.514–1.130)	0.176
Tumor stage		
Ta	1 (Reference)	n.a.
Tis/T1	1.362 (0.955–1.943)	0.088
Tumor grading		
G1	1 (Reference)	n.a.
G2	0.719 (0.456–1.135)	0.156
G3	1.519 (0.987–2.337)	0.057
Resection margins		
sR0	1 (Reference)	n.a.
sR1	2.028 (1.351–3.044)	<0.001
sRx	1.174 (0.475–2.907)	0.728
CIS		
Not detected	1 (Reference)	n.a.
Detected	1.366 (0.901–2.072)	0.142
Tumor focality		
Unifocal	1 (Reference)	n.a.
Multifocal	1.482 (1.030–2.133)	0.035
Tumor size		
≤2 cm	1 (Reference)	n.a.
>2 cm ≤4 cm	0.981 (0.612–1.574)	0.938
>4 cm ≤6 cm	0.761 (0.383–1.511)	0.435
>6 cm	1.251 (0.172–9.084)	0.825
Instillation therapy		
None	1 (Reference)	n.a.
BCG	0.526 (0.288–0.964)	0.038
Mitomycin	0.764 (0.528–1.108)	0.156
**Multivariate Model for Overall Survival**		
Parameter	HR (95% CI)	*p* value
Procedure		
Conventional	1 (Reference)	n.a.
PDD	1.247 (0.727–2.141)	0.423
Age, years	1.072 (1.045–1.100)	<0.001
Sex		
Female	1 (Reference)	n.a.
Male	0.776 (0.455–1.322)	0.351
Tumor stage		
Ta	1 (Reference)	n.a.
Tis/T1	0.991 (0.551–1.784)	0.977
Tumor Grading		
G1	1 (Reference)	n.a.
G2	0.971 (0.473–1.604)	0.657
G3	2.082 (0.998–4.347)	0.051
Resection margins		
sR0	1 (Reference)	n.a.
sR1	2.538 (1.431–4.502)	0.001
sRx	0.830 (0.192–3.587)	0.802
CIS		
Not detected	1 (Reference)	n.a.
Detected	0.520 (0.255–1.060)	0.072
Tumor focality		
Unifocal	1 (Reference)	n.a.
Multifocal	1.474 (0.903–2.407)	0.121
Tumor size		
≤2 cm	1 (Reference)	n.a.
>2 cm ≤4 cm	1.087 (0.655–1.804)	0.747
>4 cm ≤6 cm	0.825 (0.383–1.778)	0.624
>6 cm	1.070 (0.111–10.387)	0.953
Instillation therapy		
None	1 (Reference)	n.a.
BCG	0.535 (0.220–1.299)	0.167
Mitomycin	0.902 (0.541–1.504)	0.692

**Table 4:** Univariate and multivariate models for recurrence-free survival.

Univariate Models for Recurrence-Free Survival		
Parameter	HR (95% CI)	*p* value
Procedure		
Conventional	1 (Reference)	n.a.
PDD	0.424 (0.314–0.573)	<0.001
Age, years	0.988 (0.976–0.999)	0.045
Sex		
Female	1 (Reference)	n.a.
Male	1.220 (0.865–1.721)	0.257
Tumor stage		
Ta	1 (Reference)	n.a.
Tis/T1	1.025 (0.765–1.373)	0.867
Tumor grading		
G1	1 (Reference)	n.a.
G2	1.240 (0.888–1.732)	0.208
G3	0.866 (0.588–1.275)	0.466
Resection margins		
sR0	1 (Reference)	n.a.
sR1	1.260 (0.878–1.807)	0.210
sRx	0.517 (0.228–1.170)	0.113
CIS		
Not detected	1 (Reference)	n.a.
Detected	1.236 (0.798–1.582)	0.504
Tumor focality		
Unifocal	1 (Reference)	n.a.
Multifocal	0.861 (0.629–1.178)	0.349
Tumor size		
≤2 cm	1 (Reference)	n.a.
>2 cm ≤4 cm	0.654 (0.407–1.050)	0.079
>4 cm ≤6 cm	0.916 (0.500–1.679)	0.777
>6 cm	<0.001 (0–Inf)	0.996
Instillation therapy		
None	1 (Reference)	n.a.
BCG	1.038 (0.679–1.588)	0.863
Mitomycin	1.156 (0.848–1.575)	0.359
**Multivariate Model for Recurrence-Free Survival**		
Parameter	HR (95% CI)	*p* value
Procedure		
Conventional	1 (Reference)	n.a.
PDD	0.432 (0.263–0.708)	<0.001
Age, years	1.000 (0.982–1.019)	0.965
Sex		
Female	1 (Reference)	n.a.
Male	0.937 (0.545–1.611)	0.815
Tumor stage		
Ta	1 (Reference)	n.a.
Tis/T1	0.645 (0.361–1.157)	0.138
Tumor Grading		
G1	1 (Reference)	n.a.
G2	1.566 (0.898–2.729)	0.114
G3	1.262 (0.590–2.699)	0.548
Resection margins		
sR0	1 (Reference)	n.a.
sR1	1.326 (0.712–2.467)	0.374
sRx	0.890 (0.265–2.984)	0.850
CIS		
Not detected	1 (Reference)	n.a.
Detected	1.361 (0.720–2.573)	0.343
Tumor focality		
Unifocal	1 (Reference)	n.a.
Multifocal	1.099 (0.696–1.734)	0.687
Tumor size		
≤2 cm	1 (Reference)	n.a.
>2 cm ≤4 cm	0.632 (0.379–1.054)	0.079
>4 cm ≤6 cm	0.881 (0.466–1.665)	0.696
>6 cm	<0.001 (0–Inf)	0.996
Instillation therapy		
None	1 (Reference)	n.a.
BCG	1.296 (0.653–2.574)	0.459
Mitomycin	1.158 (0.708–1.893)	0.559

## Data Availability

The data that support the findings of this study are not publicly available because their information could compromise the privacy of research participants. However, they are available from the corresponding author upon reasonable request.

## References

[1] Sung H., Ferlay J., Siegel R.L., Laversanne M., Soerjomataram I., Jemal A., Bray F. (2021). Global Cancer Statistics 2020: GLOBOCAN Estimates of Incidence and Mortality Worldwide for 36 Cancers in 185 Countries. CA Cancer J. Clin..

[2] Burger M., Catto J.W., Dalbagni G., Grossman H.B., Herr H., Karakiewicz P., Kassouf W., Kiemeney L.A., La Vecchia C., Shariat S. (2013). Epidemiology and risk factors of urothelial bladder cancer. Eur. Urol..

[3] Babjuk M., Burger M., Compérat E.M., Gontero P., Mostafid A.H., Palou J., van Rhijn B.W.G., Rouprêt M., Shariat S.F., Sylvester R. (2019). European Association of Urology Guidelines on Non-muscle-invasive Bladder Cancer (TaT1 and Carcinoma In Situ)—2019 Update. Eur. Urol..

[4] Chang S.S., Boorjian S.A., Chou R., Clark P.E., Daneshmand S., Konety B.R., Pruthi R., Quale D.Z., Ritch C.R., Seigne J.D. (2016). Diagnosis and Treatment of Non-Muscle Invasive Bladder Cancer: AUA/SUO Guideline. J. Urol..

[5] Yang H., Lin J., Gao P., He Z., Kuang X., Li X., Fu H., Du D. (2020). Is the En Bloc Transurethral Resection More Effective than Conventional Transurethral Resection for Non-Muscle-Invasive Bladder Cancer? A Systematic Review and Meta-Analysis. Urol. Int..

[6] Mariappan P., Finney S.M., Head E., Somani B.K., Zachou A., Smith G., Mishriki S.F., N’Dow J., Grigor K.M. (2012). Good quality white-light transurethral resection of bladder tumours (GQ-WLTURBT) with experienced surgeons performing complete resections and obtaining detrusor muscle reduces early recurrence in new non-muscle-invasive bladder cancer: Validation across time and place and recommendation for benchmarking. BJU Int..

[7] Mariappan P., Zachou A., Grigor K.M. (2010). Detrusor muscle in the first, apparently complete transurethral resection of bladder tumour specimen is a surrogate marker of resection quality, predicts risk of early recurrence, and is dependent on operator experience. Eur. Urol..

[8] Shoshany O., Mano R., Margel D., Baniel J., Yossepowitch O. (2014). Presence of detrusor muscle in bladder tumor specimens—Predictors and effect on outcome as a measure of resection quality. Urol. Oncol..

[9] Sylvester R.J., van der Meijden A.P., Oosterlinck W., Witjes J.A., Bouffioux C., Denis L., Newling D.W., Kurth K. (2006). Predicting recurrence and progression in individual patients with stage Ta T1 bladder cancer using EORTC risk tables: A combined analysis of 2596 patients from seven EORTC trials. Eur. Urol..

[10] Sylvester R.J., Rodríguez O., Hernández V., Turturica D., Bauerová L., Bruins H.M., Bründl J., van der Kwast T.H., Brisuda A., Rubio-Briones J. (2021). European Association of Urology (EAU) Prognostic Factor Risk Groups for Non-muscle-invasive Bladder Cancer (NMIBC) Incorporating the WHO 2004/2016 and WHO 1973 Classification Systems for Grade: An Update from the EAU NMIBC Guidelines Panel. Eur. Urol..

[11] Brausi M., Collette L., Kurth K., van der Meijden A.P., Oosterlinck W., Witjes J.A., Newling D., Bouffioux C., Sylvester R.J. (2002). Variability in the recurrence rate at first follow-up cystoscopy after TUR in stage Ta T1 transitional cell carcinoma of the bladder: A combined analysis of seven EORTC studies. Eur. Urol..

[12] Baltacı S., Bozlu M., Yıldırım A., Gökçe M., Tinay İ., Aslan G., Can C., Türkeri L., Kuyumcuoğlu U., Mungan A. (2015). Significance of the interval between first and second transurethral resection on recurrence and progression rates in patients with high-risk non-muscle-invasive bladder cancer treated with maintenance intravesical Bacillus Calmette-Guérin. BJU Int..

[13] Grimm M.O., Steinhoff C., Simon X., Spiegelhalder P., Ackermann R., Vogeli T.A. (2003). Effect of routine repeat transurethral resection for superficial bladder cancer: A long-term observational study. J. Urol..

[14] Soria F., Giordano A., Gontero P. (2020). Transurethral resection of bladder tumor and the need for re-transurethral resection of bladder tumor: Time to change our practice?. Curr. Opin. Urol..

[15] Kakiashvili D.M., van Rhijn B.W., Trottier G., Jewett M.A., Fleshner N.E., Finelli A., Azuero J., Bangma C.H., Vajpeyi R., Alkhateeb S. (2011). Long-term follow-up of T1 high-grade bladder cancer after intravesical bacille Calmette-Guérin treatment. BJU Int..

[16] Kausch I., Sommerauer M., Montorsi F., Stenzl A., Jacqmin D., Jichlinski P., Jocham D., Ziegler A., Vonthein R. (2010). Photodynamic diagnosis in non-muscle-invasive bladder cancer: A systematic review and cumulative analysis of prospective studies. Eur. Urol..

[17] Burger M., Stief C.G., Zaak D., Stenzl A., Wieland W.F., Jocham D., Otto W., Denzinger S. (2009). Hexaminolevulinate is equal to 5-aminolevulinic acid concerning residual tumor and recurrence rate following photodynamic diagnostic assisted transurethral resection of bladder tumors. Urology.

[18] Suarez-Ibarrola R., Hein S., Farin E., Waldbillig F., Kriegmair M.C., Ritter M., Klingler H.C., Herrmann T.R.W., Gratzke C., Miernik A. (2020). Current Standards in the Endoscopic Management of Bladder Cancer: A Survey Evaluation among Urologists in German-Speaking Countries. Urol. Int..

[19] Richterstetter M., Wullich B., Amann K., Haeberle L., Engehausen D.G., Goebell P.J., Krause F.S. (2012). The value of extended transurethral resection of bladder tumour (TURBT) in the treatment of bladder cancer. BJU Int..

[20] Mannan H.R. (2017). A practical Application of a simple bootstrapping method for assessing predictors selected for epidemiologic risk models using automated variable selection. Int. J. Stat. Appl..

[21] Akand M., Muilwijk T., Raskin Y., De Vrieze M., Joniau S., Van Der Aa F. (2019). Quality Control Indicators for Transurethral Resection of Non-Muscle-Invasive Bladder Cancer. Clin. Genitourin. Cancer.

[22] Kim H.S., Jeong C.W., Kwak C., Kim H.H., Ku J.H. (2019). Novel nomograms to predict recurrence and progression in primary non-muscle-invasive bladder cancer: Validation of predictive efficacy in comparison with European Organization of Research and Treatment of Cancer scoring system. World J. Urol..

[23] Lu M., Chen S., Zhou Q., Wang L., Peng T., Wang G. (2019). Predicting recurrence of nonmuscle-invasive bladder cancer (Ta-T1): A study based on 477 patients. Medicine.

[24] Miyake M., Matsuyama H., Teramukai S., Kinoshita F., Yokota I., Matsumoto H., Shimada K., Kinjyo M., Shimokama T., Okumura K. (2020). A new risk stratification model for intravesical recurrence, disease progression, and cancer-specific death in patients with non-muscle invasive bladder cancer: The J-NICE risk tables. Int. J. Clin. Oncol..

[25] Sylvester R.J., Oosterlinck W., Holmang S., Sydes M.R., Birtle A., Gudjonsson S., De Nunzio C., Okamura K., Kaasinen E., Solsona E. (2016). Systematic Review and Individual Patient Data Meta-analysis of Randomized Trials Comparing a Single Immediate Instillation of Chemotherapy After Transurethral Resection with Transurethral Resection Alone in Patients with Stage pTa-pT1 Urothelial Carcinoma of the Bladder: Which Patients Benefit from the Instillation?. Eur. Urol..

[26] Taoka R., Matsuoka Y., Kohashiguchi K., Miura T., Tohi Y., Miyauchi Y., Kato T., Tsunemori H., Ueda N., Kakehi Y. (2020). Impact of second transurethral resection on recurrence in patients with high-grade Ta bladder cancer. Int. J. Urol..

[27] Mowatt G., N’Dow J., Vale L., Nabi G., Boachie C., Cook J.A., Fraser C., Griffiths T.R. (2011). Photodynamic diagnosis of bladder cancer compared with white light cystoscopy: Systematic review and meta-analysis. Int. J. Technol. Assess. Health Care.

[28] Stenzl A., Burger M., Fradet Y., Mynderse L.A., Soloway M.S., Witjes J.A., Kriegmair M., Karl A., Shen Y., Grossman H.B. (2010). Hexaminolevulinate guided fluorescence cystoscopy reduces recurrence in patients with nonmuscle invasive bladder cancer. J. Urol..

[29] Gallagher K.M., Gray K., Anderson C.H., Lee H., Stewart S., Donat R., Mariappan P. (2017). ‘Real-life experience’: Recurrence rate at 3 years with Hexvix(^®^) photodynamic diagnosis-assisted TURBT compared with good quality white light TURBT in new NMIBC-a prospective controlled study. World J. Urol..

[30] Veeratterapillay R., Gravestock P., Nambiar A., Gupta A., Aboumarzouk O., Rai B., Vale L., Heer R. (2021). Time to Turn on the Blue Lights: A Systematic Review and Meta-analysis of Photodynamic Diagnosis for Bladder Cancer. Eur. Urol. Open. Sci..

[31] Capece M., Spirito L., La Rocca R., Napolitano L., Buonopane R., Di Meo S., Sodo M., Bracale U., Longo N., Palmieri A. (2020). Hexaminolevulinate blue light cystoscopy (Hal) assisted transurethral resection of the bladder tumour vs white light transurethral resection of the bladder tumour in non-muscle invasive bladder cancer (NMIBC): A retrospective analysis. Arch. Ital. Urol. Androl..

[32] Miyake M., Nishimura N., Nakai Y., Fujii T., Owari T., Hori S., Morizawa Y., Gotoh D., Anai S., Torimoto K. (2021). Photodynamic Diagnosis-Assisted Transurethral Resection Using Oral 5-Aminolevulinic Acid Decreases the Risk of Repeated Recurrence in Non-Muscle-Invasive Bladder Cancer: A Cumulative Incidence Analysis by the Person-Time Method. Diagnostics.

[33] Rolevich A.I., Zhegalik A.G., Mokhort A.A., Minich A.A., Vasilevich V.Y., Polyakov S.L., Krasny S.A., Sukonko O.G. (2017). Results of a prospective randomized study assessing the efficacy of fluorescent cystoscopy-assisted transurethral resection and single instillation of doxorubicin in patients with non-muscle-invasive bladder cancer. World J. Urol..

